# Genomic landscape of antimicrobial resistance in India: findings from a multi-species surveillance study

**DOI:** 10.1038/s44259-026-00185-9

**Published:** 2026-02-16

**Authors:** Nazneen Gheewalla, Vasundhara Karthikeyan, Yuvraj Jadhav, Kirti Kulkarni, Akansha Tyagi, Jaisri Jagannadham, Sandeep Budhiraja, Bansidhar Tarai, Maithili Kavathekar, Shraddha Karve

**Affiliations:** 1 Trivedi School of Biosciences, Ashoka University, Sonipat, India; 2 George-August-Universitat, Gottingen, Germany; 3 Biological E. Limited, Hyderabad, India; 4 Sahyadri Hospitals Pvt Ltd, Pune, Maharashtra India; 5 Max Super Speciality Hospital (A unit of Devki Devi Foundation), Saket, New Delhi India; 6 Institute of Internal Medicine, Max Super Speciality Hospital (A unit of Devki Devi Foundation), Saket, New Delhi India; 7 Max Healthcare, New Delhi, India; 8 Koita Centre for Digital Health, Ashoka University, Sonipat, India

**Keywords:** Antibiotics, Genomics

## Abstract

Antimicrobial resistance (AMR) is a major public health threat, especially in low- and middle-income countries (LMICs), where large datasets linking antimicrobial susceptibility testing (AST) with genomic data remain limited. We analyzed AST results and whole genomes from 266 resistant bacterial isolates representing diverse species and specimen sources, collected from Northern and Western India between 2022 and 2024. Correlation of genomic resistance predictions with AST data revealed an overprediction of resistance by genomic methods. To our knowledge, this is the first study to systematically examine these discrepancies across multiple antibiotic-pathogen combinations in India and to identify promising targets for genomic resistance prediction. We also investigated the predominant antibiotic resistance genes (ARGs), plasmids, and other mobile genetic elements associated with them. Overall, our findings contribute meaningfully to the genomic epidemiology of AMR in India and support the development of molecular diagnostics for antimicrobial resistance.

## Introduction

Antimicrobial resistance (AMR) is a major public health challenge involving several pathogens affecting diverse organs/organ systems. Rapid evolution and spread of resistance demand better prevention, limiting transmission as well as swift development of new drugs. The last decade has seen a significant increase in efforts on all these fronts. For those efforts to be successful, however, we need comprehensive surveillance of AMR, including an extensive knowledge of AMR’s genomic landscape. This need is urgent in the low and middle-income countries (LMICs) that typically have a greater burden of AMR and limited means for surveillance^1^.

Many LMICs, including India, have AMR surveillance that primarily relies on antibiotic susceptibility testing (AST) data from tertiary healthcare centres^2–5^. In some cases, it is supplemented with genomic data about the resistance-conferring gene^6,7^. However, these approaches are far from comprehensive in resistance tracking, making it insufficient for addressing broader questions, such as: What are the most prevalent antibiotic resistance genes (ARGs) for a particular antibiotic class? Are these ARGs uniformly distributed across different geographic regions and pathogenic species? How does the genomic landscape of AMR in India compare to the global patterns?

To effectively answer these questions, a deeper understanding of the genomic basis of resistance is required, which can be achieved through whole-genome sequencing (WGS) of diverse AMR pathogens. Previously, several studies have reported WGS data from clinical isolates in India. The majority, however, focus on specific pathogens, analyzing sequence types (STs), ARGs, and, in some cases, associated plasmids^8,9^. *K. pneumoniae*, *S. typhi*, and *E. coli* are the most-sequenced pathogenic species in India^10^. Even for these pathogens, the extent of available WGS data varies widely across different geo-climatic zones of India with majority of the sequences representing diversity from the southern geo-climatic zone^10^. A recent study examined the genomic landscape of AMR across multiple pathogens in India^11^. The findings emphasize the pathogen-specific nature of ARGs, resistance allele heterogeneity and geo-specificity of Indian isolates when compared with global lineages^11^. These observations underscore the need for continued genomic surveillance through WGS in India.

Beyond the need for ongoing surveillance, several critical questions remain unanswered. For instance:

How accurately can ARG-based predictions determine resistance for different pathogen-drug combinations? This is crucial for developing molecular diagnostic tools but remains under-researched. Another key question is whether specific ARGs are primarily plasmid-associated, influencing their transferability between species and the speed of resistance spread. Addressing these gaps is essential for enhancing AMR surveillance, guiding diagnostics, and informing resistance control strategies.

In this work, we attempt to fill this gap to an extent by sequencing 266 isolates of the priority pathogens collected from Northern and Western India over the period of two years. We study the genomic underpinnings of carbapenem, colistin, vancomycin and methicillin resistance in India. We also look into the association of ARGs with either plasmids or chromosomes. Notably, we address the question of whether resistance predictions based on genomics match the AST for all drug-pathogen combinations.

## Results

### Epidemiological characteristics and the resistance categories of the sequenced isolates

**Fig. 1:**
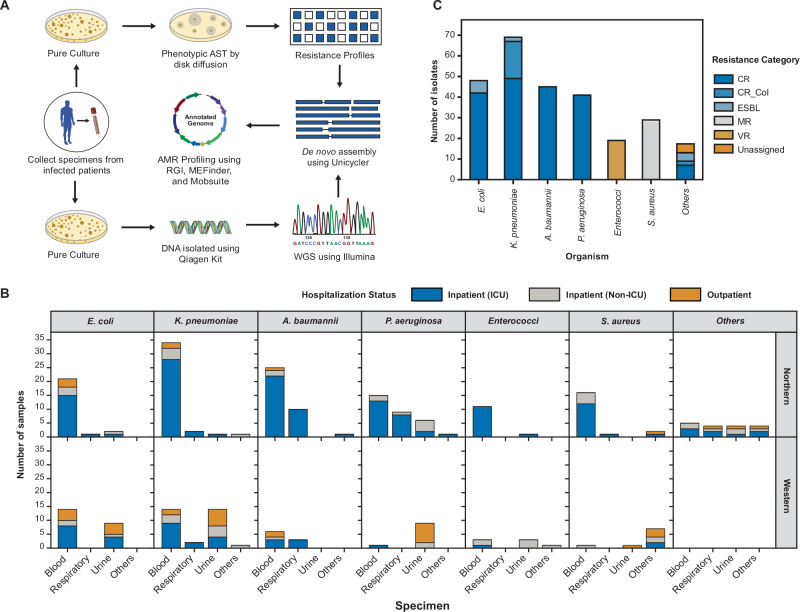
Study workflow and summary of metadata. **A** Overview of the study design and bioinformatic pipeline. We collected bacterial isolates from a variety of clinical specimens and performed antimicrobial susceptibility testing (AST) and whole-genome sequencing. We generated draft genome assemblies and used standard bioinformatic tools to assess the prevalence of antibiotic resistance genes (ARGs) and their associations with mobile genetic elements. **B** Geographic and clinical distribution of isolates by species. Each panel displays the number of isolates for a given species across two regions of India (Northern and Western) and three specimen types: blood, urine, and others. Bar colours represent patient hospitalization status: dark blue for ICU inpatients, light blue for non-ICU inpatients, and orange for outpatients. **C** Distribution of sequenced isolates by six AST-derived resistance categories (as indicated in the legend): CR (carbapenem-resistant), CR_Col (carbapenem and colistin-resistant), ESBL (extended-spectrum β-lactam-resistant), MR (methicillin-resistant), VR (vancomycin-resistant) and unassigned. The x-axis shows bacterial species, while the y-axis indicates the number of isolates per species. Most sequenced isolates were Gram-negative pathogens from four species: *Escherichia coli*, *Klebsiella pneumoniae*, *Acinetobacter baumannii* and *Pseudomonas aeruginosa*. The unassigned represents two isolates of *B. cepacia* and one isolate of *S. pneumoniae* that do not belong to any of the other five resistance categories.

We analyzed 266 bacterial genomes from the two groups of tertiary healthcare centres, one in Northern and the other in Western India. We selected isolates for sequencing based on culture sensitivity criteria (Methods). After DNA extraction at the hospital labs, we performed short-read sequencing, de novo assembly, annotation, and downstream analysis (Fig. 1A, Methods). The study included priority pathogens such as *K. pneumoniae*, *E. coli*, *A. baumannii*, *P. aeruginosa*, *S. aureus*, and *E. faecium*, as well as other emerging threats like *P. mirabilis*, *B. cepacia*, *E. cloacae*, *P. rettgeri*, and *S. pneumoniae*^2,12^. We sequenced several Carbapenem-resistant (CR) gram-negative pathogens, with *K. pneumoniae* (49 isolates) being the most sequenced (Fig. 1B). We sequenced 20 carbapenem-colistin-resistant (CR-Col) isolates and 12 β-lactam-resistant but carbapenem-sensitive gram-negative isolates. We also included Methicillin-resistant *S. aureus* (MRSA, 28 isolates) and vancomycin-resistant *Enterococcus* (VRE, 19 isolates) (Fig. 1B).

Of the 266 isolates, 177 were from Northern India and 89 from Western India (Fig. 1B). Specimen sources included blood (166, comprising 62.4%), urine (50), respiratory samples (32), and other sources like pus and body fluids (18) (Fig. 1B). Patient distribution spanned outpatient (38), non-ICU wards (51), and ICU wards (177). The dataset was diverse in demographics, with 105 isolates from female and 161 from male patients, covering a broad age range, predominantly over 40 years (Supplementary data 1).

Our dataset captures diverse bacterial genomes from distinct geographic regions and resistance categories, forming a foundation for understanding the molecular basis of antibiotic resistance in India.

### Agreement between culture sensitivity reports and resistance prediction using genomic data

For genomic AMR predictions, we used the Resistance Gene Identifier (RGI) tool, associated with the Comprehensive Antibiotic Resistance Database (CARD), that offers several advantages over other databases^13,14^. We assessed the agreement between RGI predictions and AST results across 266 isolates from 12 species and 56 antibiotics. Among 5437 comparisons, 592 discrepancies emerged.

**Fig. 2:**
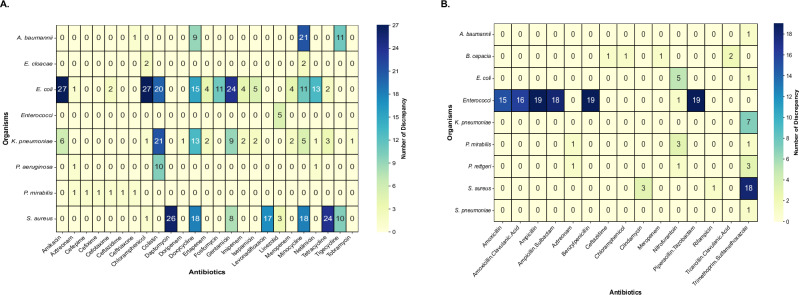
Discrepancies between phenotypic (antibiotic susceptibility testing, AST) and genotypic (RGI-CARD) predictions of antibiotic resistance across 5437 drug-pathogen combinations. The horizontal axis represents antibiotics, while the vertical axis lists bacterial species. Colour intensity ranges from light yellow (no discrepancies) to dark blue (high number of discrepancies). Drugs or bacterial species with no observed discrepancies are not shown in the heatmap. **A** Major discrepancies, where AST indicated susceptibility, but RGI predicted resistance. **B** Very major discrepancies, where AST indicated resistance that was not detected by RGI.

Most (433) were ‘major discrepancies’, where RGI predicted resistance, but cultures showed sensitivity (Fig. 2A). Minocycline had the highest (57) major discrepancies across all the isolates. Other antibiotics like colistin, doxycycline, gentamicin and tigecycline also frequently displayed ‘major’ discrepancies. Amongst the bacterial species, *E. coli* accounted for the most ‘major’ discrepancies (170 of 433 instances). ‘Very major’ discrepancies (159 cases) are those when AST indicated resistance, but RGI did not (Fig. 2B). The highest very major discrepancies involved trimethoprim-sulfamethoxazole (~13%). *Enterococci* contributed to over 65% of very major discrepant cases, particularly for β-lactam drugs like amoxicillin and ampicillin.

A few of the observed discrepancies were present in the clinically important drug-pathogen combinations (Supplementary data 2). For instance, RGI predicted sensitivity against ampicillin and ampicillin-sulbactam for several *E. faecium* isolates (Fig. 1B) that showed resistance in the AST. In another case, 11 uropathogenic *E. coli* were predicted to be resistant to fosfomycin by RGI but were seen to be sensitive by AST (Fig. 1A). Colistin also exhibited several major discrepancies. In some cases of major discrepancies, it was difficult to determine whether the correct prediction was made by phenotypic or genomic assessment. For example, 21 *A. baumannii* isolates were predicted to be resistant to minocycline by RGI but were categorized as sensitive in AST. Sixteen of these 21 isolates harboured the AdeABC efflux pump, which is known to raise the MIC for minocycline but is intrinsic to *A. baumannii* and does not affect bacterial killing at CLSI breakpoint concentrations of the antibiotic^15^. However, five of the 21 isolates also showed the *tetB* gene when the cut-off for detection was extended to “strict” in CARD_RGI. This cut-off is expected to detect variants of the *tetB* gene that are very similar to known resistance-conferring variants. Recent PK-PD studies have shown that the presence of the *tetB* gene can render *A. baumannii* isolates, that are classified as susceptible by CLSI guidelines, non-susceptible to minocycline^16^.

Overall, RGI predictions aligned reasonably well with AST data, with most discrepancies being conservative (‘major’).

### Genomic determinants of resistance to β-lactam antibiotics

Of the 266 isolates sequenced, 202 belonged to four priority pathogenic species: *E. coli* (47), *K. pneumoniae* (69), *A. baumannii* (45), and *P. aeruginosa* (41). β-lactam antibiotics, which inhibit peptidoglycan synthesis, are the primary treatment for these pathogens. We detected several ARGs, including efflux pumps, mutant penicillin-binding proteins (PBPs), and porin variants (Supplementary table 1), but we focused on beta-lactamase genes due to their impact on therapeutic management.

**Fig. 3:**
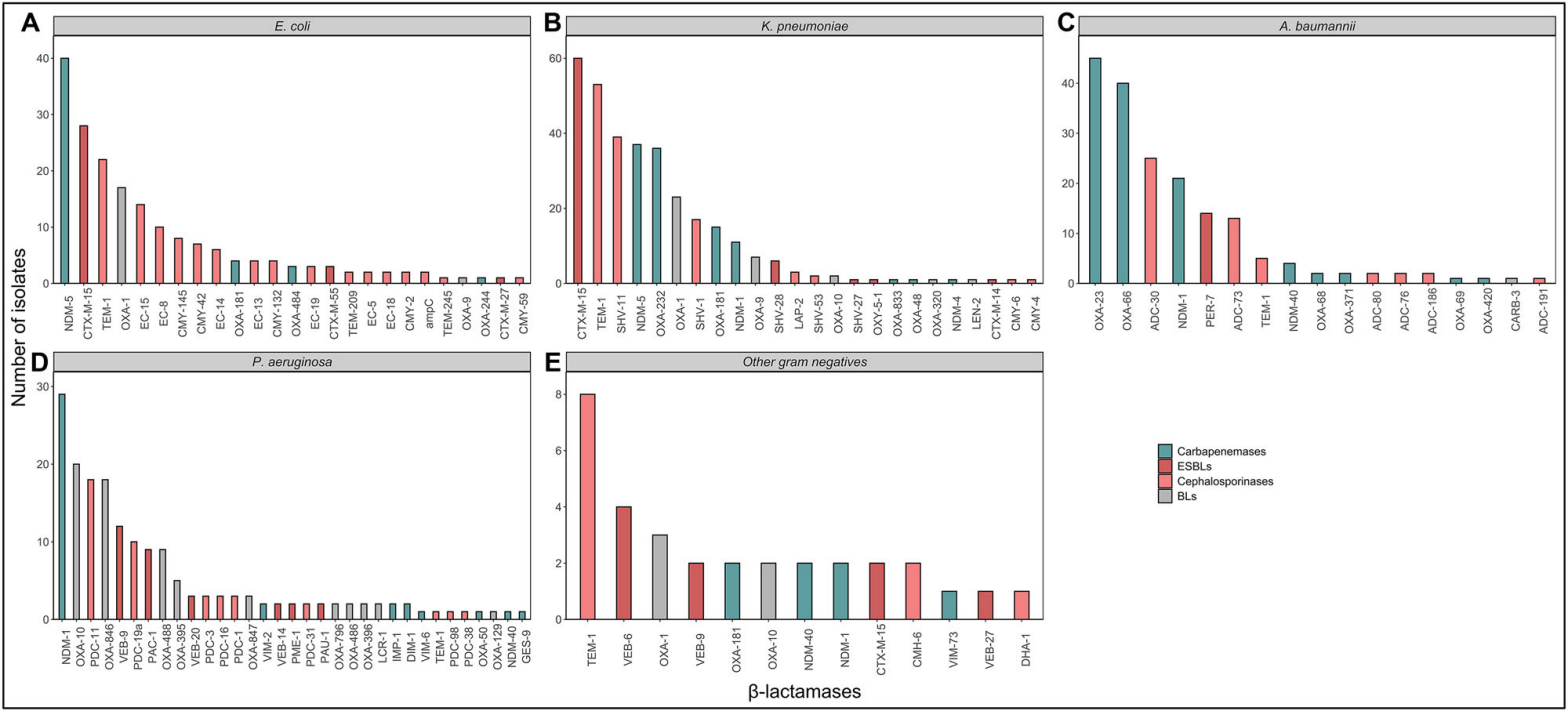
Prevalence of β-lactamases among 213 Gram-negative isolates. Each bar on the x-axis represents the number of isolates from a given species carrying a particular β-lactamase gene. The colour of the bars denotes the β-lactamase category based on their substrate profiles and classes as indicated by CARD-RGI, Bacterial Antimicrobial Resistance Reference Gene Database and Bush and Jacoby, 2010^17^^–^^19^. Supplementary table 2 provides the detailed information on the criteria for gene classification and exceptional cases. In the figure, green represents carbapenemases, dark red shows extended-spectrum β-lactamases (ESBLs), pink shows non-ESBL cephalosporinases, and grey represents narrow-spectrum β-lactamases. The panels display the distribution for: **A** *Escherichia coli*, **B** *Klebsiella pneumoniae*, **C** *Acinetobacter baumannii*, **D** *Pseudomonas aeruginosa* **E**. Other Gram-negative species (*Providencia rettgeri*, *Proteus mirabilis*, *Burkholderia cepacia*, *Enterobacter cloacae*).

We identified at least one β-lactamase gene in all 202 isolates, with 87 distinct genes across 865 occurrences, spanning 24 gene families (Supplementary table 2)^17^. We classified the genes as carbapenemases, extended spectrum β-lactamases (ESBLs), the ampC β-lactamases and other narrow spectrum cephalosporinases (cephalosporinases) and narrow spectrum β-lactamases (BLs)^17–19^. There were genes for 22 carbapenemases, 14 ESBLs, 36 non-ESBL cephalosporinases and 15 BLs (Fig. 3).

We detected 305 occurrences of carbapenemases in 185 isolates. Metallo-β-lactamases *bla*_NDM-5_ (77 isolates) and *bla*_NDM-1_ (61 isolates) were the most common. *bla*_NDM-1_ was present in *P. aeruginosa* (70.7% isolates), *A. baumannii* (46.6% isolates) and *K. pneumoniae* (16% isolates). *bla*_NDM-5_ was present in over 53% of *K. pneumoniae* and 85% of *E. coli* isolates. We detected other metallo-β-lactamases, *bla*_NDM-40_ (4*A. baumannii*, 1 *P. aeruginosa*) and *bla*_NDM-4_ (1 *K. pneumoniae*). *P. aeruginosa* carried additional carbapenemase genes, including *bla*_DIM_, *bla*_IMP_, *bla*_VIM_, and *bla*_GES_ (Fig. 3D). We detected 14 carbapenemase-encoding *bla*_OXA_ genes with *A. baumannii* isolates exhibiting the highest diversity (Fig. 3C).

At least one ESBL-encoding gene was present in 138 isolates, with 145 total instances. *bla*_CTX-M_ genes were most prevalent and exclusive to *Enterobacteriaceae*, with *bla*_CTX-M-15_ being the most prevalent variant (88 isolates). The other two species harboured their own unique ESBLs, such as *bla*_PER-7_ in *A. baumanii* and *bla*_VEB_, *bla*_PAC_, *bla*_PAU_, *bla*_PME_ in *P. aeruginosa*. Other ESBLs found included *bla*_SHV-27_, *bla*_SHV-28_, *bla*_OXY-5-1_.

In non-ESBL cephalosporinases, *bla*_TEM-1_ was the most prevalent variant, found in *K. pneumoniae* (53 isolates), *E. coli* (22 isolates), *A. baumanii* (5 isolates) and *P. aeruginosa* (1 isolate). The *bla*_SHV_ family was exclusive to *K. pneumoniae*, with *bla*_SHV-11_ (39 isolates) and *bla*_SHV-1_ (17 isolates) being the most common. Other cephalosporinases included *bla*_SHV-1_, *bla*_SHV-11_ and extended-spectrum ampC (ESAC) beta-lactamases like *bla*_PDC_ variants and *bla*_ADC_ variants. Several *ampC* genes that are typically plasmid-mediated, such as *bla*_*CMY*_ variants, were present^20,21^. *E. coli* exclusively carried the intrinsic *bla*_EC_ genes, with *bla*_EC-15_ and *bla*_EC-8_ being the most common^22^.

We also detected several genes encoding BLs. Most notable beta-lactamases in this category include the *bla*_OXA_ variants such as *bla*_OXA-1_ in *E. coli* (17 isolates) and *K. pneumoniae* (23 isolates), *bla*_OXA-10_ in *P. aeruginosa* (20 isolates) and *bla*_OXA-846_ in *P. aeruginosa* (18 isolates). Other BLs included *bla*_LEN-2_ (*K. pneumoniae*), *bla*_CARB-3_ (*A. baumannii*) and *bla*_LCR-1_ (*P. aeruginosa*) (Fig. 3).

Beyond the four priority pathogens that we sequenced extensively, we detected several β-lactamases in 11 isolates from other gram-negative species: *bla*_NDM-1_, *bla*_NDM-40_, *bla*_OXA-181_, *bla*_VIM-73_, *bla*_TEM-1_, *bla*_CTX-M-15_, *bla*_VEB-9_, *bla*_VEB-6_, *bla*_DHA-1_, *bla*_OXA-1_, *bla*_CMH-6_, *bla*_OXA-10_ and *bla*_VEB-27_ (Fig. 3E). In the Gram-positives, most *S. aureus* isolates (25 of 28) carried *bla*_Z_, conferring penicillin resistance.

### Genomic determinants of Colistin, Vancomycin, and Methicillin resistance

Among 266 isolates, 247 carried peptide antibiotic resistance genes, including 32 unique variants, 27 of which were linked to colistin resistance^23^. Of the 214 isolates with colistin-associated genes tested via AST, resistance was confirmed in 18 *K. pneumoniae*, and 2 *E. cloacae* isolates. All *Proteus* (7) and *Providencia* (3) isolates were intrinsically resistant. Among the remaining 184, 115 had intermediate sensitivity, while 69 were sensitive. In the AST-resistant *K. pneumoniae* and *E. cloacae* isolates, we detected several peptide-resistance conferring genes, including *arnT, pmrF* (*arnC*), *kpnE*, and *kpnF*. Surprisingly, the globally significant plasmid-based *mcr* (*mcr9.1*) gene was found in only 2 *E. cloacae* isolates (Supplementary data 3)^23,24^. We noted several ‘major’ discrepancies with colistin where RGI predicted resistance for phenotypically sensitive isolates (Fig. 2A).

To understand the genomic determinants of methicillin resistance in *S. aureus*, we sequenced 28 MRSAs. All 28 isolates harboured the *mecA* gene, which confers resistance to methicillin. One MRSA isolate also carried the mecR1 regulator gene. *mgrA*, a global regulator gene involved in virulence as well as efflux pump expression in *S. aureus*, was also present in all 28 isolates (Supplementary table 3).

For studying the genomic determinants of vancomycin resistance, we looked at the genes that confer resistance to glycopeptides, the drug class consisting of vancomycin, except the bleomycin resistance-conferring gene, *BRP*^17^. Of the 266 isolates that we sequenced, 221 isolates carried at least one glycopeptide resistance gene (Supplementary data 4). We looked at the *Enterococcus* (19), *S. pneumoniae* (1) and *S. aureus* (28) isolates that are commonly treated with the glycopeptide drugs and were also tested for vancomycin resistance in AST. All enterococci were resistant to vancomycin in AST and harboured the entire *vanA*-gene cluster (7 genes); 11 of these isolates also harboured the *cfr(D)* gene. Other Gram-positive isolates (AST-sensitive) showed the presence of single *van* genes but not the entire clusters (Supplementary data 4).

### Sequence types and associated ARGs

We used Multilocus Sequence Typing (MLST) to further characterize the sequenced isolates and their molecular epidemiology (Supplementary data 1). PubMLST failed to assign any sequence type (ST) to two *K. pneumoniae*, five *P. aeruginosa*, three *A. baumannii*, seven *P. mirabilis*, three *P. rettgeri* and three *S. aureus* isolates. We further explored the common STs and associated ARGs for the four pathogenic species with a relatively large number of sequenced isolates.

*E. coli* displayed the highest sequence type diversity, with 17 unique STs. Among the 47 *E. coli* isolates that we sequenced, two were known epidemic clones commonly found in India: ST167 (12 isolates) and ST131 (4 isolates)^25^. ST131 is also globally infamous for causing urinary tract and bloodstream infections in inpatient settings^26,27^. Three out of four ST131 isolates carried *bla*_*CTX-M-15*_. Other prominent sequence types in *E. coli* were ST405 (7) and ST410 (5). Consistent with previous reports^25^, we detected *bla*_*NDM-5*_ in several isolates, including but not limited to ST167, ST131, ST410, and ST405. Apart from β-lactamases, the sulfonamide resistance-conferring gene *sul1* was also present in over 80% of isolates belonging to ST167, ST405, ST131 and ST410.

Among *K. pneumoniae* isolates, we detected some known prevalent sequence types such as ST147 (19 of 69) and ST16 (7 of 69), while other expected sequence types, such as ST14, were absent^28^. Both ST147 and ST16 showed the presence of *sul1* in over 85% of isolates. Additionally, all ST147 isolates harboured *bla*_*SHV-11*_, while only one of the seven ST16 isolates carried this gene. All seven ST16 isolates harboured *bla*_*CTX-M-15*_ and *bla*_*TEM-1*_. Other common sequence types for *K. pneumoniae* included ST231 (9), ST395 (7), and ST437 (7). The most commonly found *bla*_*OXA*_ gene, *bla*_*OXA-232*_, was associated with ST147, ST231, ST395, and ST437, whereas *bla*_*OXA-181*_ was more commonly associated with ST16.

*P. aeruginosa* isolates showed limited sequence type diversity, with eight unique STs among the 36 isolates that were successfully typed by PubMLST. ST357, a widespread sequence type in India^29^, was the most prevalent sequence type in our data with 18 isolates. This was followed by ST308 with nine isolates. All ST357 isolates carried the *bla*_*OXA-846*_ and intrinsic *bla*_*PDC-11*_, while all ST308 isolates carried intrinsic *bla*_*PDC-19a*_^22,30^ along with the sulfonamide resistance-conferring gene *sul2*.

Out of the 45*A. baumannii* isolates that we sequenced, 37 belonged to ST2. All ST2 isolates carried intrinsic *bla*_*OXA-23*_ and *bla*_*OXA-66*_^22,30^, while over 50% were also associated with *bla*_*NDM-1*_. The sulfonamide resistance-conferring genes *sul1* and *sul2* were present in over 60% of ST2 isolates. All but one ST2 isolates also carried the macrolide resistance-conferring gene *mphE*.

In the four pathogenic species, we observed substantial sequence type diversity in *E. coli* and *K. pneumoniae*, and relatively lower diversity in *A. baumannii* and *P. aeruginosa*. Although some β-lactamase–encoding genes showed strong associations with specific STs, we note that the number of isolates for any particular species was limited in our study. This limitation prevents us from making solid inferences about sequence type prevalences or sequence type–ARG associations.

### Patterns of distribution, diversity and ARG associations for plasmids and mobile genetic elements

We next determined whether detected ARGs were plasmid-borne or chromosomal, as plasmids play a key role in AMR dissemination. From the 266 isolates, we identified 1,400 predicted plasmids using MOB-suite v3.1.9^31^, primarily from *E. coli, K. pneumoniae*, and *A. baumannii* (Supplementary table 4).

**Fig. 4:**
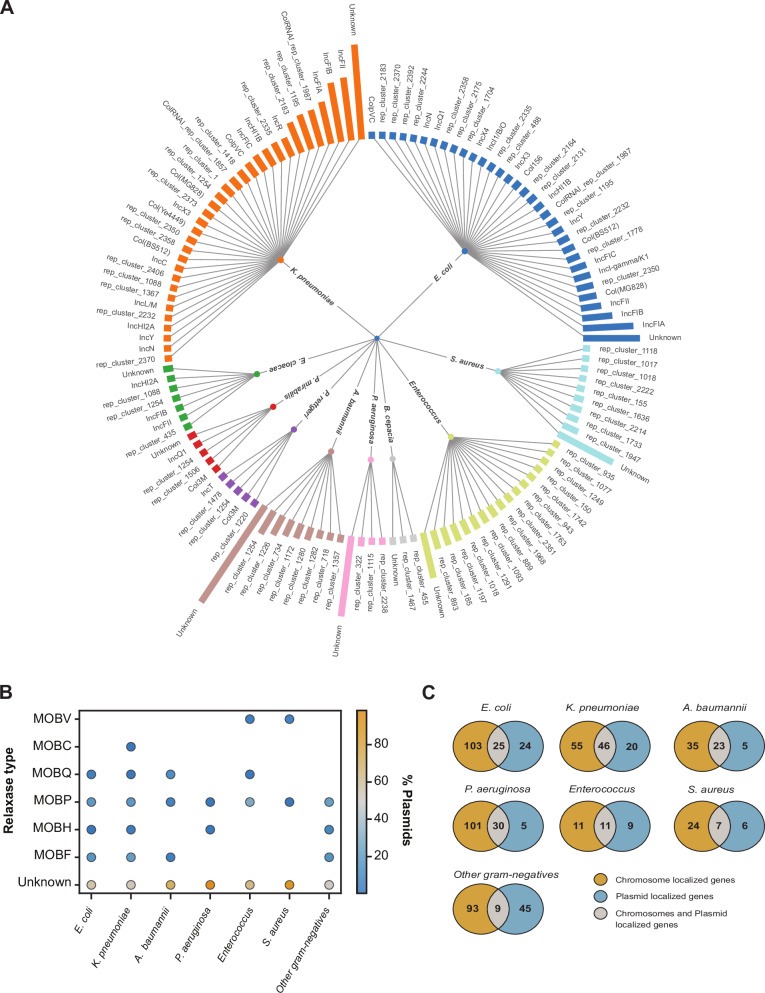
Overview of plasmid replicon diversity, relaxase distribution across bacterial species. **A** Radial dendrogram showing plasmid replicons across species. Each colour represents a species and length of the bar denotes the frequency. *K. pneumoniae* represents the highest diversity, followed by *E. coli* (**B**). Bubble plot displaying the distribution of MOB relaxase types across species. Colour intensity represents the occurrence with orange colour denoting high occurrence. *P. aeruginosa* and *S. aureus* harbour a large number of unknown relaxase. **C** Venn diagrams illustrating the localization of ARGs across different species. *K. pneumoniae* harbours the highest fraction of ARGs on plasmids or both on the plasmid and chromosome.

Plasmid clustering revealed high diversity in *E. coli* (261 plasmids, 104 clusters), *K. pneumoniae* (469 plasmids, 115 clusters), and *E. cloacae* (11 plasmids, 6 clusters), and much lower diversity in *A. baumannii* (250 plasmids, 25 clusters). Plasmid typing highlighted annotation gaps in *P. aeruginosa* (94.83% untyped) and *A. baumannii* (58% untyped), unlike *E. coli* and *K. pneumoniae*, with only ~20% untyped plasmids. We identified 87 unique replicon types (Fig. 4A) and six relaxase types (Fig. 4B), with single-replicon, single-relaxase plasmids dominating. *K. pneumoniae* and *E. coli* harboured numerous multi-replicon plasmids (120 and 52, respectively). Additionally, *K. pneumoniae* had 54 multi-relaxase plasmids, suggesting a strong potential for horizontal gene transfer (HGT) (Supplementary Table 4)^32^.

*E. coli* predominantly harboured IncF replicons (IncFIA, IncFIB, IncFII) (Fig. 4A) linked to multidrug resistance, followed by Col(MG828), lncl-gamma/K1, and rep_cluster_2350, which are linked to broad-host-range and widespread HGT^33,34^. In *K. pneumoniae*, dominant IncF types were IncFII, IncFIB and IncFIA, which are known to carry carbapenemases and other β-lactamases^35^. *A. baumannii* displayed limited plasmid diversity (seven replicons), with rep_cluster_1254 being most frequent. Relaxase types overlapped between species, though their distributions were species-specific: *E. coli* plasmids predominantly carried MOBP, followed by MOBF, while *K. pneumoniae* had the reverse. *A. baumannii* carried mostly MOBQ (Fig. 4B). *P. aeruginosa* plasmids had fewer relaxase annotations, yet MOBP and MOBH were detected.

We next analyzed ARG localization in the four priority species with over 40 sequenced isolates using the RGI tool^17^ (Fig. 4C, Supplementary data 5). *P. aeruginosa* exhibited the highest chromosome localization of ARGs (74%), followed by *E. coli* (66%) and *A. baumannii* (55%). *K. pneumoniae* had only 45% of ARGs on the chromosome, indicating a greater role of horizontal gene transfer (Fig. 4C). Localization preferences varied across species and ARGs. Resistance-nodulation-division (RND) efflux pump genes (*acr, ade, mdt, mar, oqx*) were almost exclusively chromosomal (>96% across species) as expected^36,37^, while major-facilitator-superfamily (MFS) efflux pump genes were predominantly chromosomal only in *E. coli* and *K. pneumoniae*. The sulfonamide resistance gene *sul1*, was plasmid-localized across all four species, whereas *sul2* preferred plasmids were only found in *E. coli*. In *S. aureus*, 22 of 35 ARGs, including *bla*_Z_ that codes for a β-lactamase and *mecA* that confers resistance to cefoxitin, were chromosomal. In contrast, vancomycin resistance genes (*vanA, vanS*) were predominantly plasmid-localized in *Enterococcus*.

Clinically significant β-lactamase genes were detected on both plasmids and chromosomes, with a bias toward plasmid localization. In *E. coli*, some β-lactamases (e.g. *CMY-42, CMY-59, PER-7*), ESBLs (e.g. *CTX-M-27, CTX-M-55*) and carbapenemases (e.g. *OXA-484, OXA-181, OXA-66*) were exclusively plasmid-localized. *K. pneumoniae* harboured *NDM-4* exclusively on plasmids. *A. baumannii* had *PER-7, OXA-420*, and *OXA-371* exclusively on plasmids. *TEM-1* was exclusively plasmid-localized in *P. aeruginosa*. Interestingly, certain ARGs also showed replicon-type preferences. In *A. baumannii*, >85% of plasmid-localized ARGs were linked to replicon cluster 1254 (Fig. 4A). Similarly, IncFIA plasmids in *E. coli* frequently carried β-lactamase, sulfonamide, and tetracycline resistance genes. Many ARGs were associated with unclassified plasmids, including 133 instances in *A. baumannii* and nearly all plasmid-localized ARGs in *P. aeruginosa* (Supplementary data 5).

### Mobile genetic elements and associated ARGs

**Fig. 5:**
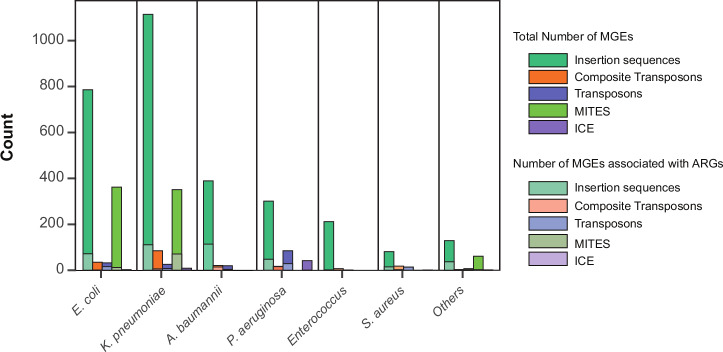
Types of mobile genetic elements. Grouped stacked bar plot showing total and ARG-associated MGEs, categorized by type. Other Gram-negatives include *P. mirabilis*, *P. rettgeri*, *E. cloacae*, and *B. cepacia*. *E. coli* and *K. pneumoniae* show a large number of MGEs while *A. baumanii* shows the highest fraction of ARG-associated MGE.

ARGs can spread between bacteria through mobile genetic elements (MGEs), regardless of whether they are on chromosomes or plasmids. To investigate this, we examined the prevalence of different MGEs such as insertion sequences (IS), unit transposons (Tn), miniature inverted-repeat transposable elements (MITEs), integrative and conjugative elements (ICEs), and composite transposons (Supplementary data 6) using MobileElementFinder v1.1.2^38^. *K. pneumoniae* had the highest MGE burden, with 1,114 IS elements (111 ARG-associated), 26 transposons (8 ARG-associated), 351 MITEs (71 ARG-associated), and 85 composite transposons (6 ARG-associated) (Fig. 5). Interestingly, *A. baumannii*, despite having fewer MGEs, had a higher proportion linked to ARGs, with 114 of its 389 IS elements associated with resistance (Fig. 5). Notably, macrolide resistance genes were among the most frequently associated with MGEs across multiple species.

Different types of MGEs varied in numbers and localization preferences across species. For example, plasmid-borne IS elements were abundant in *K. pneumoniae*, but evenly distributed between chromosomes and plasmids in *E. coli*. MITEs, however, were almost exclusively found on chromosomes in all the species. Putative composite transposons—comprising two identical or similar IS elements flanking an AMR gene-containing region—were abundant in *K. pneumoniae*, *P. aeruginosa*, and *A. baumannii* but not in others (Supplementary data 6).

## Discussion

We report whole-genome sequences and AST data for 266 priority pathogens, examining the correlation between genomic resistance predictions and AST, the genetic determinants of key resistance categories, and the association of ARGs with plasmids and chromosomes to assess transmissibility.

One of the key features of our work is the assessment of the genomic predictions of resistance in comparison with the observed AST across diverse pathogenic species. Our discrepancy analysis showed that the genomic method overpredicted the resistance (Fig. 2A). We chose CARD-RGI for its conservative approach to predicting AMR. The tool uses empirical evidence to estimate increases in MIC, which are interpreted as indicators of resistance. However, this predicted increase in MIC could be of any magnitude and may not render the pathogen resistant at the breakpoint concentration as per CLSI (or EUCAST) guidelines.

CARD-RGI’s disregard for the CLSI (or EUCAST) breakpoints also means that genomic predictions of resistance do not distinguish between the intrinsic and acquired resistance genes. The degree of resistance conferred by intrinsic genes is inadequate to breach the clinical breakpoints. For instance, all our *K. pneumoniae* isolates harboured some variant of *bla*_*SHV*_ gene that was flagged by CARD-RGI (Supplementary data 7). Other β-lactamase genes that are intrinsic to other pathogens, such as *bla*_*EC*_ in *E. coli, bla*_*ADC*_ *in A. baumanii* and *bla*_*PDC*_ in *P. aeruginosa*, also resulted in genomically predicted resistance^22,30^. Similar was the case with other core chromosomal genes, such as Acr efflux pump genes in *E. coli* and Ade efflux pump genes in *A. baumanii*^36,37^.

Nevertheless, the false positive predictions of resistance by genomic methods should not be ignored entirely for a couple of reasons. First, they indicate that the isolate has increased MICs and may become resistant at the clinical breakpoint concentrations. Second, the false positive predictions reveal the important gaps in the knowledge. Accurate genomic predictions of resistance can revolutionize patient outcomes, especially in critical care units like ICUs. For instance, we report 11 instances of overprediction of resistance by RGI to fosfomycin in uropathogenic *E. coli*, 5 of which were from ICUs. In fact, more than 66% of the 266 isolates that we sequenced were from ICUs, where rapid prediction through molecular diagnostics can be extremely valuable. Therefore, the gaps identified in our study are crucial to address to enhance prediction accuracy.

Plasmid diversity analysis also revealed large gaps in the existing genomic datasets. For instance, we found very little data on plasmid annotations in *P. aeruginosa* (Fig. 4A). Other cases of many missing annotations, such as *S. aureus* and *A. baumannii*, were also unique to our study, indicating the diversity of plasmid backbones, including cryptic plasmids, exceeds the current capabilities of available typing schemes^39^. For the identified plasmid backbones, we studied the ARG-associations. As expected, we discovered many ARGs to be plasmid-associated (Fig. 4C). We also detected many known intrinsic and acquired ARGs on the chromosome. For instance, Ade pump components were found to be chromosomally located in *A. baumanii* isolates and Acr efflux pump components were exclusively chromosomal in *E. coli* and *Klebsiella* isolates^36,40^. With the available annotation data, we show that no pathogen relied solely on either chromosomal or plasmid-localized ARGs for resistance to a specific antibiotic class. This complicates strategies to reduce ARG prevalence by limiting antibiotic use, as chromosomal ARGs may persist even when plasmid-associated ones diminish.

A key limitation of our study is uneven sampling across species, specimens, and regions, constrained by logistical challenges. This prevented rigorous analysis of resistance as well as any meaningful conclusions by sequence typing of isolates (Supplementary data 1). Additionally, some species in the “Other” category had very few isolates, which prevented any solid analysis (Fig. 1B). Another limitation is our reliance on short-read sequencing technologies, which, while robust and cost-effective, have definite drawbacks^41^. For instance, we detected rep_cluster_455 in *B. cepacia* isolates, a broad-range replicon found in *Klebsiella*, *Serratia*, and *Laribacter*, with a predicted host range spanning *Pseudomonadota*^31^. However, fragmented short-read assemblies prevent confirmation of its presence and origin. Integrating long-read sequencing could help address these challenges. We can also explore associations of ARG with prophages with in-depth analysis of viral hallmark genes and structural modules in future work^42^.

A classical surveillance study typically focuses on a species and prevalence of resistance in it^8,9^. In a departure from this strategy, we chose a broad surveillance approach that included isolates from multiple species and priority resistance categories. This allowed us to uncover the diversity of genomic determinants within a particular resistance category. For example, though we found *K. pneumoniae* isolates carried *bla*_NDM-1_ as previously reported, *bla*_NDM-5_ was much more abundant^11^. Such variations could stem from targeted sampling, evolving resistance patterns, or regional differences, but they highlight the need for extensive genomic surveillance that can result in a comprehensive genomic database of ARG variants in India. Extensive genomic surveillance can accelerate the research for molecular diagnostic tools and treatment options by allowing rapid in silico testing of candidates^43^.

## Methods

### Selection of clinical isolates

We collected 266 bacterial isolates between July 2022 and July 2024 from two groups of tertiary healthcare centres, one in Northern and the other in Western India. We obtained ethical approvals for genomic sequencing and de-identified metadata collection. We selected isolates from diverse specimens (Fig. 1C, Supplementary data 1) based on the priority lists published by the Indian Council for Medical Research and the Department of Biotechnology^2,12^. We included high-priority pathogenic groups of carbapenem-resistant *E. coli* (CREC), carbapenem-resistant *K. pneumoniae* (CRKP), carbapenem-resistant *P. aeruginosa* (CRPA) and carbapenem-resistant *A. baumannii* (CRAB); methicillin-resistant *S. aureus* (MRSA) and Vancomycin-resistant Enterococci (VRE), along with extended-spectrum beta-lactamase-producing *E. coli* (ESBL *E. coli*), ESBL-positive *K. pneumoniae*. We also included a few pathogenic species that are acquiring resistance at an alarming rate and are likely to pose a challenge in the near future. This category included *B. cepacia, E. cloacae, P. mirabilis, P. rettgeri* and *S. pneumoniae*. We classified the selected isolates into six resistance categories: Carbapenem-resistant (CR), Carbapenem & colistin-resistant (CR_Col), Extended spectrum β-lactam resistant (ESBL), Methicillin-resistant (MR), Vancomycin-resistant (VR) and unassigned (Fig. 1B). We considered relevant categories for every species (e.g., MR for *S. aureus;* CR, ESBL, and CR_Col for *E. coli*, *K. pneumoniae* etc).

In case of Enterobacteriaceae such as *E. coli* and *K. pneumoniae*, we considered resistance to either one or more of ertapenem, imipenem or meropenem as CR, while for non-fermenting gram-negative bacteria (NFNGB) such as *A. baumannii* and *P. aeruginosa*, resistance to either imipenem or meropenem (or both) was considered as CR as per Indian Council of Medical Research (ICMR) guidelines. ESBL positivity was determined as resistance to at least one or more third generation cephalosporin and to aztreonam. We considered resistance to cefoxitin or oxacillin as a proxy for detecting methicillin resistance of *S. aureus* as per Clinical and Laboratory Standards Institute (CLSI) guidelines. Four isolates had unique resistance profiles and could not be grouped under either of the above categories. These include three *B. cepacia* isolates and the only *S. pneumoniae* isolate.

Prior to AST and genomic sequencing, we used VITEK 2 (bioMérieux), VITEK-MS (bioMérieux) or standard biochemical tests for species identification. We collected antibiotic susceptibility test reports (henceforth, AST) and metadata (Supplementary data 1).

We stored the isolates as glycerol stocks at -80°C and revived them in tryptone soy media. We excluded the isolates with insufficient growth, contamination, or morphological changes after revival.

### Isolate culture, Genomic DNA extraction, Library preparation and Sequencing protocols

We stored the isolates as glycerol stocks at -80°C. We revived the isolates on McConkey’s or Blood agar and excluded the isolates with insufficient growth, contamination, or morphological changes post revival. We suspended the revived culture in water for injection to ~1.2 × 10⁹ cfu/ml (verified via densitometer, McFarland 4 standard), and processed using the Qiagen Blood & Tissue Kit (Cat No: 69504). Gram-positive bacteria underwent additional lysis with lysozyme (0.5-1.5 mg) before extraction. We eluted the DNA in 10 mM Tris-Cl (pH 8, by Merck or GeNei) and quantified using Qubit and Nanodrop. We sequenced the extracted DNA using the Illumina platform. We outsourced sequencing to two different sequencing facilities: Eurofins Genomics, Bangalore, India and National Centre for Biological Sciences (NCBS), Bangalore, India. Short-read sequencing was performed using the Illumina NextSeq 500/NovaSeq 6000 platforms. Libraries were prepared using the Illumina TruSeq Nano DNA Library Prep Kit or Illumina DNA Prep Kit (Tagmentation). ~200 ng of extracted DNA was fragmented to obtain 350 bp long fragments using either an ultrasonicator (Covaris M220) or tagmentation. Fragments were subjected to end-repair, adaptor ligation, size selection and PCR amplification. After confirming the quality of the prepared libraries using the Agilent 4200 Tapestation, the quality-passing libraries were sequenced on the Illumina NextSeq 500 or NovaSeq 6000 Platform with the corresponding read length of 2 x 150 bp and 2 x 100 bp respectively.

### Genomic assembly and downstream analysis

We quality-checked the raw reads using FastQC v0.12.1 before and after trimming. We trimmed the adapter, quality-filtered the raw reads using Trimmomatic v0.39 and built de novo assemblies using Unicycler v0.5.0 in the normal mode^44–46^. We evaluated the assemblies against the 3 C criterion: the quality and contiguity using QUAST v5.2.0, completeness using the BUSCO and correctness using BWA v0.7.17^47–50^. We checked the completeness and contamination using Checkm2 v1.1.0^51^. All 266 genomic assemblies showed the completeness of 99% or above. The contamination score was <1% for 224 genomic assemblies. 41 genomes showed between 1 to 5% contamination, while only one *E. faecium* isolate showed 6% contamination. CheckM results for all the 266 isolates are provided as Supplementary data 8.

We annotated the assemblies using Prokka v1.14.5^52^. We identified antimicrobial resistance genes using the locally installed RGI v6.0.3 (‘Perfect’ and ‘Strict’ cut-offs)^17^. The list of all detected ARGs for all the isolates is provided as Supplementary Dataset_ARG. We reconstructed and annotated the plasmids using MOB-suite v3.1.9 and used MobileElementFinder v1.1.2 to identify the mobile elements and RGI v6.0.3 to identify the resistance genes associated with them^17,31,38^. We considered a resistance gene to be associated with the identified MGEs if they are present within the 30 Kb region of an MGE. We used the PubMLST database and the associated MLST v2.23.0 tool for the Multilocus Sequence Typing of the isolates^53^. Sequence types for all the typed isolates are provided in the Supplementary data 1.

We used R (v4.5.0) and Python (v3.12) for writing custom-made scripts for the analysis and data visualization.

### Agreement between AST and genomic predictions

Following a previous study, we analyzed two discrepancy conditions^14^. One instance where AST indicated sensitivity, but RGI predicted resistance using a ‘Perfect’ RGI cut-off. We classified this condition as ‘a major’ discrepancy. Two instances where AST indicated resistance, but RGI predicted sensitivity using ‘Perfect’ and ‘Strict’ cut-offs (i.e., including strongly suspected resistance-conferring gene variants along with the well-established ones), a ‘very major’ discrepancy.

## Supplementary information

Supplementary material

S data 1

S data 2

S data 3

S data 4

S data 5

S data 6

S data 7

S data 8

## Data Availability

Sequencing data has been uploaded on NCBI under the Bioproject PRJNA1273658. We have now provided the review link as a hyperlink in the data statement. https://dataview.ncbi.nlm.nih.gov/object/PRJNA1273658?reviewer=c3itcfann33ffems6gpjfd0b4e.
