# Supplementary material for: Genomic landscape of antimicrobial resistance in India: findings from a multi-species surveillance study
Source: NPJ Antimicrob Resist. 2026 Feb 16;4:13. doi: 10.1038/s44259-026-00185-9 (PMC12910060; doi:10.1038/s44259-026-00185-9)
Supplement: Supplementary file 1 — Supplementary material [file 44259_2026_185_MOESM1_ESM.pdf]

Supplementary material:

1. **Supplementary table 1:**
   List of genes other than beta-lactamases that can confer resistance to beta-lactam antibiotics and the number of isolates from each species which show presence of the gene.

Organism	Gene	Gene Family	Number of isolates with gene presence
A. baumannii	*adeI*	resistance-nodulation-cell division (RND) antibiotic efflux pump	45
A. baumannii	*adeJ*	resistance-nodulation-cell division (RND) antibiotic efflux pump	45
A. baumannii	*adeK*	resistance-nodulation-cell division (RND) antibiotic efflux pump	45
A. baumannii	*adeN*	resistance-nodulation-cell division (RND) antibiotic efflux pump	25
E. cloacae	*CRP*	resistance-nodulation-cell division (RND) antibiotic efflux pump	2
E. cloacae	*Enterobacter cloacae acrA*	resistance-nodulation-cell division (RND) antibiotic efflux pump	2
E. cloacae	*Escherichia coli AcrAB-TolC with MarR mutations conferring resistance to ciprofloxacin and tetracycline*	resistance-nodulation-cell division (RND) antibiotic efflux pump	2
E. cloacae	*Escherichia coli soxS with mutation conferring antibiotic resistance*	ATP-binding cassette (ABC) antibiotic efflux pump; major facilitator superfamily (MFS) antibiotic efflux pump; resistance-nodulation-cell division (RND) antibiotic efflux pump; General Bacterial Porin with reduced permeability to beta-lactams	2
E. cloacae	*Haemophilus influenzae PBP3 conferring resistance to beta-lactam antibiotics*	Penicillin-binding protein mutations conferring resistance to beta-lactam antibiotics	2
E. cloacae	*Klebsiella pneumoniae KpnE*	small multidrug resistance (SMR) antibiotic efflux pump	2
E. cloacae	*Klebsiella pneumoniae KpnF*	small multidrug resistance (SMR) antibiotic efflux pump	2
E. cloacae	*acrB*	resistance-nodulation-cell division (RND) antibiotic efflux pump	2
E. cloacae	*marA*	resistance-nodulation-cell division (RND) antibiotic efflux pump; General Bacterial Porin with reduced permeability to beta-lactams	2
E. cloacae	*ramA*	resistance-nodulation-cell division (RND) antibiotic efflux pump; General Bacterial Porin with reduced permeability to beta-lactams	2
E. coli	*AcrE*	resistance-nodulation-cell division (RND) antibiotic efflux pump	35
E. coli	*AcrF*	resistance-nodulation-cell division (RND) antibiotic efflux pump	46
E. coli	*AcrS*	resistance-nodulation-cell division (RND) antibiotic efflux pump	35
E. coli	*CRP*	resistance-nodulation-cell division (RND) antibiotic efflux pump	47
E. coli	*Escherichia coli AcrAB-TolC with AcrR mutation conferring resistance to ciprofloxacin, tetracycline, and ceftazidime*	resistance-nodulation-cell division (RND) antibiotic efflux pump	38
E. coli	*Escherichia coli AcrAB-TolC with MarR mutations conferring resistance to ciprofloxacin and tetracycline*	resistance-nodulation-cell division (RND) antibiotic efflux pump	47
E. coli	*Escherichia coli acrA*	resistance-nodulation-cell division (RND) antibiotic efflux pump	47
E. coli	*Escherichia coli soxR with mutation conferring antibiotic resistance*	ATP-binding cassette (ABC) antibiotic efflux pump; major facilitator superfamily (MFS) antibiotic efflux pump; resistance-nodulation-cell division (RND) antibiotic efflux pump	47
E. coli	*Escherichia coli soxS with mutation conferring antibiotic resistance*	ATP-binding cassette (ABC) antibiotic efflux pump; major facilitator superfamily (MFS) antibiotic efflux pump; resistance-nodulation-cell division (RND) antibiotic efflux pump; General Bacterial Porin with reduced permeability to beta-lactams	47
E. coli	*H-NS*	major facilitator superfamily (MFS) antibiotic efflux pump; resistance-nodulation-cell division (RND) antibiotic efflux pump	47
E. coli	*Haemophilus influenzae PBP3 conferring resistance to beta-lactam antibiotics*	Penicillin-binding protein mutations conferring resistance to beta-lactam antibiotics	47
E. coli	*Klebsiella pneumoniae KpnE*	small multidrug resistance (SMR) antibiotic efflux pump	47
E. coli	*Klebsiella pneumoniae KpnF*	small multidrug resistance (SMR) antibiotic efflux pump	47

E. coli	*TolC*	ATP-binding cassette (ABC) antibiotic efflux pump; major facilitator superfamily (MFS) antibiotic efflux pump; resistance-nodulation-cell division (RND) antibiotic efflux pump	47
E. coli	*acrB*	resistance-nodulation-cell division (RND) antibiotic efflux pump	47
E. coli	*evgA*	major facilitator superfamily (MFS) antibiotic efflux pump; resistance-nodulation-cell division (RND) antibiotic efflux pump	46
E. coli	*evgS*	major facilitator superfamily (MFS) antibiotic efflux pump; resistance-nodulation-cell division (RND) antibiotic efflux pump	45
E. coli	*gadW*	resistance-nodulation-cell division (RND) antibiotic efflux pump	33
E. coli	*gadX*	resistance-nodulation-cell division (RND) antibiotic efflux pump	47
E. coli	*marA*	resistance-nodulation-cell division (RND) antibiotic efflux pump; General Bacterial Porin with reduced permeability to beta-lactams	47
E. coli	*mdtE*	resistance-nodulation-cell division (RND) antibiotic efflux pump	47
E. coli	*mdtF*	resistance-nodulation-cell division (RND) antibiotic efflux pump	47
K. pneumoniae	*CRP*	resistance-nodulation-cell division (RND) antibiotic efflux pump	69
K. pneumoniae	*Escherichia coli AcrAB-TolC with MarR mutations conferring resistance to ciprofloxacin and tetracycline*	resistance-nodulation-cell division (RND) antibiotic efflux pump	69
K. pneumoniae	*Haemophilus influenzae PBP3 conferring resistance to beta-lactam antibiotics*	Penicillin-binding protein mutations conferring resistance to beta-lactam antibiotics	69
K. pneumoniae	*Klebsiella pneumoniae KpnE*	small multidrug resistance (SMR) antibiotic efflux pump	69
K. pneumoniae	*Klebsiella pneumoniae KpnF*	small multidrug resistance (SMR) antibiotic efflux pump	69
K. pneumoniae	*Klebsiella pneumoniae KpnG*	major facilitator superfamily (MFS) antibiotic efflux pump	68
K. pneumoniae	*Klebsiella pneumoniae KpnH*	major facilitator superfamily (MFS) antibiotic efflux pump	67
K. pneumoniae	*Klebsiella pneumoniae OmpK37*	General Bacterial Porin with reduced permeability to beta-lactams	68
K. pneumoniae	*Klebsiella pneumoniae ramR mutants*	resistance-nodulation-cell division (RND) antibiotic efflux pump	1
K. pneumoniae	*LptD*	ATP-binding cassette (ABC) antibiotic efflux pump	69
K. pneumoniae	*MdtQ*	Outer Membrane Porin (Opr)	68
K. pneumoniae	*Shigella flexneri acrA*	resistance-nodulation-cell division (RND) antibiotic efflux pump	64
K. pneumoniae	*acrB*	resistance-nodulation-cell division (RND) antibiotic efflux pump	69
K. pneumoniae	*marA*	resistance-nodulation-cell division (RND) antibiotic efflux pump; General Bacterial Porin with reduced permeability to beta-lactams	69
P. mirabilis	*CRP*	resistance-nodulation-cell division (RND) antibiotic efflux pump	7
P. mirabilis	*Haemophilus influenzae PBP3 conferring resistance to beta-lactam antibiotics*	Penicillin-binding protein mutations conferring resistance to beta-lactam antibiotics	7
P. mirabilis	*Klebsiella pneumoniae KpnH*	major facilitator superfamily (MFS) antibiotic efflux pump	7
P. rettgeri	*CRP*	resistance-nodulation-cell division (RND) antibiotic efflux pump	3
P. rettgeri	*Haemophilus influenzae PBP3 conferring resistance to beta-lactam antibiotics*	Penicillin-binding protein mutations conferring resistance to beta-lactam antibiotics	3
P. rettgeri	*Klebsiella pneumoniae KpnH*	major facilitator superfamily (MFS) antibiotic efflux pump	3
P. aeruginosa	*ArmR*	resistance-nodulation-cell division (RND) antibiotic efflux pump	13
P. aeruginosa	*MexA*	resistance-nodulation-cell division (RND) antibiotic efflux pump	41
P. aeruginosa	*MexB*	resistance-nodulation-cell division (RND) antibiotic efflux pump	41
P. aeruginosa	*MexC*	resistance-nodulation-cell division (RND) antibiotic efflux pump	41
P. aeruginosa	*MexD*	resistance-nodulation-cell division (RND) antibiotic efflux pump	41
P. aeruginosa	*MexR*	resistance-nodulation-cell division (RND) antibiotic efflux pump	41
P. aeruginosa	*MexZ*	resistance-nodulation-cell division (RND) antibiotic efflux pump	40
P. aeruginosa	*MuxA*	resistance-nodulation-cell division (RND) antibiotic efflux pump	41
P. aeruginosa	*MuxB*	resistance-nodulation-cell division (RND) antibiotic efflux pump	41
P. aeruginosa	*MuxC*	resistance-nodulation-cell division (RND) antibiotic efflux pump	41
P. aeruginosa	*OpmB*	resistance-nodulation-cell division (RND) antibiotic efflux pump	41

P. aeruginosa	*OprJ*	resistance-nodulation-cell division (RND) antibiotic efflux pump	41
P. aeruginosa	*OprM*	resistance-nodulation-cell division (RND) antibiotic efflux pump	41
P. aeruginosa	*ParR*	resistance-nodulation-cell division (RND) antibiotic efflux pump; Outer Membrane Porin (Opr)	41
P. aeruginosa	*ParS*	resistance-nodulation-cell division (RND) antibiotic efflux pump; Outer Membrane Porin (Opr)	40
P. aeruginosa	*Pseudomonas aeruginosa CpxR*	resistance-nodulation-cell division (RND) antibiotic efflux pump	41
P. aeruginosa	*Pseudomonas aeruginosa soxR*	ATP-binding cassette (ABC) antibiotic efflux pump; major facilitator superfamily (MFS) antibiotic efflux pump; resistance-nodulation-cell division (RND) antibiotic efflux pump	41
P. aeruginosa	*Type B NfxB*	resistance-nodulation-cell division (RND) antibiotic efflux pump	41
P. aeruginosa	*YajC*	resistance-nodulation-cell division (RND) antibiotic efflux pump	41
P. aeruginosa	*mexP*	resistance-nodulation-cell division (RND) antibiotic efflux pump	41
P. aeruginosa	*mexQ*	resistance-nodulation-cell division (RND) antibiotic efflux pump	41
P. aeruginosa	*mexY*	resistance-nodulation-cell division (RND) antibiotic efflux pump	39
P. aeruginosa	*nalC*	resistance-nodulation-cell division (RND) antibiotic efflux pump	40
P. aeruginosa	*nalD*	resistance-nodulation-cell division (RND) antibiotic efflux pump	13
P. aeruginosa	*opmE*	resistance-nodulation-cell division (RND) antibiotic efflux pump	41
S. aureus	*mecA*	methicillin resistant PBP2	28
S. aureus	*mecR1*	methicillin resistant PBP2	1
S. aureus	*mgrA*	ATP-binding cassette (ABC) antibiotic efflux pump; major facilitator superfamily (MFS) antibiotic efflux pump	28

2. **Supplementary table 2:**
   List of beta-lactamases genes and their classification along with the number of isolates from each species which show presence of the given gene.

   Note: The β-lactamase genes found across the isolates were grouped into four β-lactam categories based on their substrate profiles and classes as indicated by CARD-RGI[1], NCBI reference gene catalogue[2] and Bush and Jacoby, 2010[3] (or references therein). Few cases showed discrepancies amongst these sources and for these we referred to additional literature and grouped them accordingly (Huang et al, 2024 and Wang J et al, 2019)[4,5]. For SHV-53 we found no information regarding it's Bush and Jacoby group, thus we group it as a cephalosporinase based only on the CARD-RGI drug class data.

Organism	β-lactam category	Gene	Number of isolates with gene presence
Acinetobacter baumannii	BLs	*CARB-3*	1
Acinetobacter baumannii	Carbapenemases	*NDM-1*	21
Acinetobacter baumannii	Carbapenemases	*NDM-40*	4
Acinetobacter baumannii	Carbapenemases	*OXA-23*	45
Acinetobacter baumannii	Carbapenemases	*OXA-371*	2
Acinetobacter baumannii	Carbapenemases	*OXA-420*	1
Acinetobacter baumannii	Carbapenemases	*OXA-66*	40
Acinetobacter baumannii	Carbapenemases	*OXA-68*	2
Acinetobacter baumannii	Carbapenemases	*OXA-69*	1
Acinetobacter baumannii	Cephalosporinases	*ADC-186*	2
Acinetobacter baumannii	Cephalosporinases	*ADC-191*	1
Acinetobacter baumannii	Cephalosporinases	*ADC-30*	25
Acinetobacter baumannii	Cephalosporinases	*ADC-73*	13
Acinetobacter baumannii	Cephalosporinases	*ADC-76*	2

Acinetobacter baumannii	Cephalosporinases	*ADC-80*	2
Acinetobacter baumannii	Cephalosporinases	*TEM-1*	5
Acinetobacter baumannii	ESBLs	*PER-7*	14
Burkholderia cepacia	BLs	*OXA-10*	1
Burkholderia cepacia	Carbapenemases	*VIM-73*	1
Enterobacter cloacae	BLs	*OXA-1*	2
Enterobacter cloacae	Carbapenemases	*NDM-1*	2
Enterobacter cloacae	Cephalosporinases	*CMH-6*	2
Enterobacter cloacae	Cephalosporinases	*TEM-1*	2
Enterobacter cloacae	ESBLs	*CTX-M-15*	2
Escherichia coli	BLs	*OXA-1*	17
Escherichia coli	BLs	*OXA-9*	1
Escherichia coli	Carbapenemases	*NDM-5*	40
Escherichia coli	Carbapenemases	*OXA-181*	4
Escherichia coli	Carbapenemases	*OXA-244*	1
Escherichia coli	Carbapenemases	*OXA-484*	3
Escherichia coli	Cephalosporinases	*Escherichia coli ampC beta-lactamase (ampC)*	2
Escherichia coli	Cephalosporinases	*CMY-132*	4
Escherichia coli	Cephalosporinases	*CMY-145*	8
Escherichia coli	Cephalosporinases	*CMY-2*	2
Escherichia coli	Cephalosporinases	*CMY-42*	7
Escherichia coli	Cephalosporinases	*CMY-59*	1
Escherichia coli	Cephalosporinases	*EC-13*	4
Escherichia coli	Cephalosporinases	*EC-14*	6
Escherichia coli	Cephalosporinases	*EC-15*	14
Escherichia coli	Cephalosporinases	*EC-18*	2
Escherichia coli	Cephalosporinases	*EC-19*	3
Escherichia coli	Cephalosporinases	*EC-5*	2
Escherichia coli	Cephalosporinases	*EC-8*	10
Escherichia coli	Cephalosporinases	*TEM-1*	22
Escherichia coli	Cephalosporinases	*TEM-209*	2
Escherichia coli	Cephalosporinases	*TEM-245*	1
Escherichia coli	ESBLs	*CTX-M-15*	28
Escherichia coli	ESBLs	*CTX-M-27*	1
Escherichia coli	ESBLs	*CTX-M-55*	3
Klebsiella pneumoniae	BLs	*LEN-2*	1
Klebsiella pneumoniae	BLs	*OXA-1*	23
Klebsiella pneumoniae	BLs	*OXA-10*	2
Klebsiella pneumoniae	BLs	*OXA-320*	1
Klebsiella pneumoniae	BLs	*OXA-9*	7
Klebsiella pneumoniae	Carbapenemases	*NDM-1*	11
Klebsiella pneumoniae	Carbapenemases	*NDM-4*	1
Klebsiella pneumoniae	Carbapenemases	*NDM-5*	37
Klebsiella pneumoniae	Carbapenemases	*OXA-181*	15
Klebsiella pneumoniae	Carbapenemases	*OXA-232*	36
Klebsiella pneumoniae	Carbapenemases	*OXA-48*	1

Klebsiella pneumoniae	Carbapenemases	*OXA-833*	1
Klebsiella pneumoniae	Cephalosporinases	*CMY-4*	1
Klebsiella pneumoniae	Cephalosporinases	*CMY-6*	1
Klebsiella pneumoniae	Cephalosporinases	*LAP-2*	3
Klebsiella pneumoniae	Cephalosporinases	*SHV-1*	17
Klebsiella pneumoniae	Cephalosporinases	*SHV-11*	39
Klebsiella pneumoniae	Cephalosporinases	*SHV-53*	2
Klebsiella pneumoniae	Cephalosporinases	*TEM-1*	53
Klebsiella pneumoniae	ESBLs	*CTX-M-14*	1
Klebsiella pneumoniae	ESBLs	*CTX-M-15*	60
Klebsiella pneumoniae	ESBLs	*OXY-5-1*	1
Klebsiella pneumoniae	ESBLs	*SHV-27*	1
Klebsiella pneumoniae	ESBLs	*SHV-28*	6
Proteus mirabilis	BLs	*OXA-1*	1
Proteus mirabilis	BLs	*OXA-10*	1
Proteus mirabilis	Cephalosporinases	*DHA-1*	1
Proteus mirabilis	Cephalosporinases	*TEM-1*	4
Proteus mirabilis	ESBLs	*VEB-27*	1
Proteus mirabilis	ESBLs	*VEB-6*	4
Providencia rettgeri	Carbapenemases	*NDM-40*	2
Providencia rettgeri	Carbapenemases	*OXA-181*	2
Providencia rettgeri	Cephalosporinases	*TEM-1*	2
Providencia rettgeri	ESBLs	*VEB-9*	2
Pseudomonas aeruginosa	BLs	*LCR-1*	2
Pseudomonas aeruginosa	BLs	*OXA-10*	20
Pseudomonas aeruginosa	BLs	*OXA-129*	1
Pseudomonas aeruginosa	BLs	*OXA-395*	5
Pseudomonas aeruginosa	BLs	*OXA-396*	2
Pseudomonas aeruginosa	BLs	*OXA-486*	2
Pseudomonas aeruginosa	BLs	*OXA-488*	9
Pseudomonas aeruginosa	BLs	*OXA-796*	2
Pseudomonas aeruginosa	BLs	*OXA-846*	18
Pseudomonas aeruginosa	BLs	*OXA-847*	3
Pseudomonas aeruginosa	Carbapenemases	*DIM-1*	2
Pseudomonas aeruginosa	Carbapenemases	*GES-9*	1
Pseudomonas aeruginosa	Carbapenemases	*IMP-1*	2
Pseudomonas aeruginosa	Carbapenemases	*NDM-1*	29
Pseudomonas aeruginosa	Carbapenemases	*NDM-40*	1
Pseudomonas aeruginosa	Carbapenemases	*OXA-50*	1
Pseudomonas aeruginosa	Carbapenemases	*VIM-2*	2
Pseudomonas aeruginosa	Carbapenemases	*VIM-6*	1
Pseudomonas aeruginosa	Cephalosporinases	*PDC-1*	3
Pseudomonas aeruginosa	Cephalosporinases	*PDC-11*	18
Pseudomonas aeruginosa	Cephalosporinases	*PDC-16*	3
Pseudomonas aeruginosa	Cephalosporinases	*PDC-19a*	10
Pseudomonas aeruginosa	Cephalosporinases	*PDC-3*	3

Pseudomonas aeruginosa	Cephalosporinases	*PDC-31*	2
Pseudomonas aeruginosa	Cephalosporinases	*PDC-38*	1
Pseudomonas aeruginosa	Cephalosporinases	*PDC-98*	1
Pseudomonas aeruginosa	Cephalosporinases	*TEM-1*	1
Pseudomonas aeruginosa	ESBLs	*PAC-1*	9
Pseudomonas aeruginosa	ESBLs	*PAU-1*	2
Pseudomonas aeruginosa	ESBLs	*PME-1*	2
Pseudomonas aeruginosa	ESBLs	*VEB-14*	2
Pseudomonas aeruginosa	ESBLs	*VEB-20*	3
Pseudomonas aeruginosa	ESBLs	*VEB-9*	12
Staphylococcus aureus	BLs	*PC1 (blaZ)*	25

3. Supplementary table 3: Methicillin resistance gene matrix (as detected by CARD-RGI) in MRSA isolates

Isolate ID	Organism	Resistance Category	mecA	mgrA	mecR1
SA064	*S. aureus*	MR	+	+	-
SA067	*S. aureus*	MR	+	+	-
SA068	*S. aureus*	MR	+	+	-
SA102	*S. aureus*	MR	+	+	-
SA103	*S. aureus*	MR	+	+	-
SA105	*S. aureus*	MR	+	+	-
SA106	*S. aureus*	MR	+	+	-
SA107	*S. aureus*	MR	+	+	-
SA121	*S. aureus*	MR	+	+	-
SA122	*S. aureus*	MR	+	+	-
SA123	*S. aureus*	MR	+	+	-
SA124	*S. aureus*	MR	+	+	-
SA127	*S. aureus*	MR	+	+	+
SA158	*S. aureus*	MR	+	+	-
SA160	*S. aureus*	MR	+	+	-
SA161	*S. aureus*	MR	+	+	-
SA175	*S. aureus*	MR	+	+	-
SA196	*S. aureus*	MR	+	+	-
SA197	*S. aureus*	MR	+	+	-
SABP186	*S. aureus*	MR	+	+	-
SAPP121	*S. aureus*	MR	+	+	-
SAPP138	*S. aureus*	MR	+	+	-
SAPP139	*S. aureus*	MR	+	+	-
SAPP140	*S. aureus*	MR	+	+	-
SAPP171	*S. aureus*	MR	+	+	-
SAPP172	*S. aureus*	MR	+	+	-
SAPP174	*S. aureus*	MR	+	+	-
SAUP169	*S. aureus*	MR	+	+	-

4. **Supplementary table 4:**
Summary of plasmids identified using Mob-Suite

Organism	Number of isolates	Number of identified plasmids	Number of unique Plasmid clusters	Number of Plasmids with single rep_type	Number of Plasmids with multiple rep_type	Number of Plasmids with single relaxase_type	Number of Plasmids with multiple relaxase_type	Number of Plasmids lacking only rep_type	Number of plasmids lacking only relaxase_type	Number of plasmids lacking both the annotations
A. baumannii	45	250	25	83	7	64	2	15	39	145
B. cepacia	4	4	3	2	0	1	0	0	1	2
E. cloacae	2	11	6	2	4	4	2	0	0	5
E. coli	47	249	104	117	52	91	12	14	95	51
Enterococci	19	162	31	87	16	51	4	10	58	49
K. pneumoniae	69	469	115	218	120	134	54	22	172	109
P. aeruginosa	41	116	20	6	0	2	0	0	4	110
P. mirabilis	7	7	7	3	1	2	0	0	2	3
P. rettgeri	3	7	4	5	2	4	0	0	3	0
S. aureus	28	113	27	25	8	10	0	0	23	80

5. **Supplementary Data 1: Metadata & AST results for 266 isolates**
Patient metadata and AST results obtained from hospital partners are listed for all 266 isolates.

6. **Supplementary Data 2: List of major and very major discrepancies seen in each isolate.**
List of every major and very major discrepancies as seen in each individual isolate.

7. **Supplementary Data 3: Colistin resistance gene matrix (as detected by CARD-RGI) and colistin AST for 214 isolates**
A gene matrix of colistin resistance associated genes along with colistin AST results for 214 isolates that showed presence of atleast one colistin resistance associated gene

8. **Supplementary Data 4 Vancomycin resistant (glycopeptide resistant) gene matrix and vancomycin AST results.**
A gene matrix of vancomycin (glycopeptide) resistance associated genes along with vancomycin AST results for 221 isolates that showed presence of atleast one of the associated genes.

9. **Supplementary Data 5: Plasmid & chromosome associated AMR genes.**
A list of plasmid and chromosome associated antimicrobial resistance genes (as detected by CARD-RGI) and the isolates in which they are observed.

10. **Supplementary Data 6: Mobile Elements and associated AMR genes**
A list of mobile elements and associated antimicrobial resistance genes (as detected by CARD-RGI).

11. **Supplementary Data 7: Lists of AMR genes in the 266 isolates**
Lists of AMR genes as observed by CARD-RGI in 266 isolates for each pathogenic species

12. **Supplementary Data 8: CheckM results for 266 isolates**
CheckM results for the 266 isolates and their summary table

References:

1. Alcock BP, Huynh W, Chalil R, Smith KW, Raphenya AR, Wlodarski MA, et al. CARD 2023: expanded curation, support for machine learning, and resistome prediction at the Comprehensive Antibiotic Resistance Database. Nucleic Acids Res. 2023 Jan 6;51(D1):D690–9.
2. NCBI reference gene catalogue. https://www.ncbi.nlm.nih.gov/pathogens/refgene
3. Bush Karen, Jacoby George A. Updated Functional Classification of β-Lactamases. Antimicrob Agents Chemother. 2010 Mar 1;54(3):969–76.
4. Wang J, Xu T, Ying J, et al. PAU-1, a Novel Plasmid-Encoded Ambler Class A β-Lactamase Identified in a Clinical Pseudomonas aeruginosa Isolate. Infect Drug Resist 2019; 12: 3827–34.
5. Huang J, Zhou N, Cheng Z, et al. Chromosomally located blaCMH in Enterobacter cloacae complex across human-bird-environment interfaces: A one-health perspective. Sci Total Environ 2024; 954: 176486.
